# Supplementary material for: Splice-Junction-Based Mapping of Alternative Isoforms in the Human Proteome
Source: Cell Rep. Author manuscript; Available in PMC 2020 Jan 15. (PMC6961840; doi:10.1016/j.celrep.2019.11.026)
Supplement: 3 [file NIHMS1546469-supplement-3.zip › DF2/PXD006675/CardiacFibroblast-99-Q12797-FETLEPEVSHEETEHSYHVEETDSSEPVVEDER.pdf]

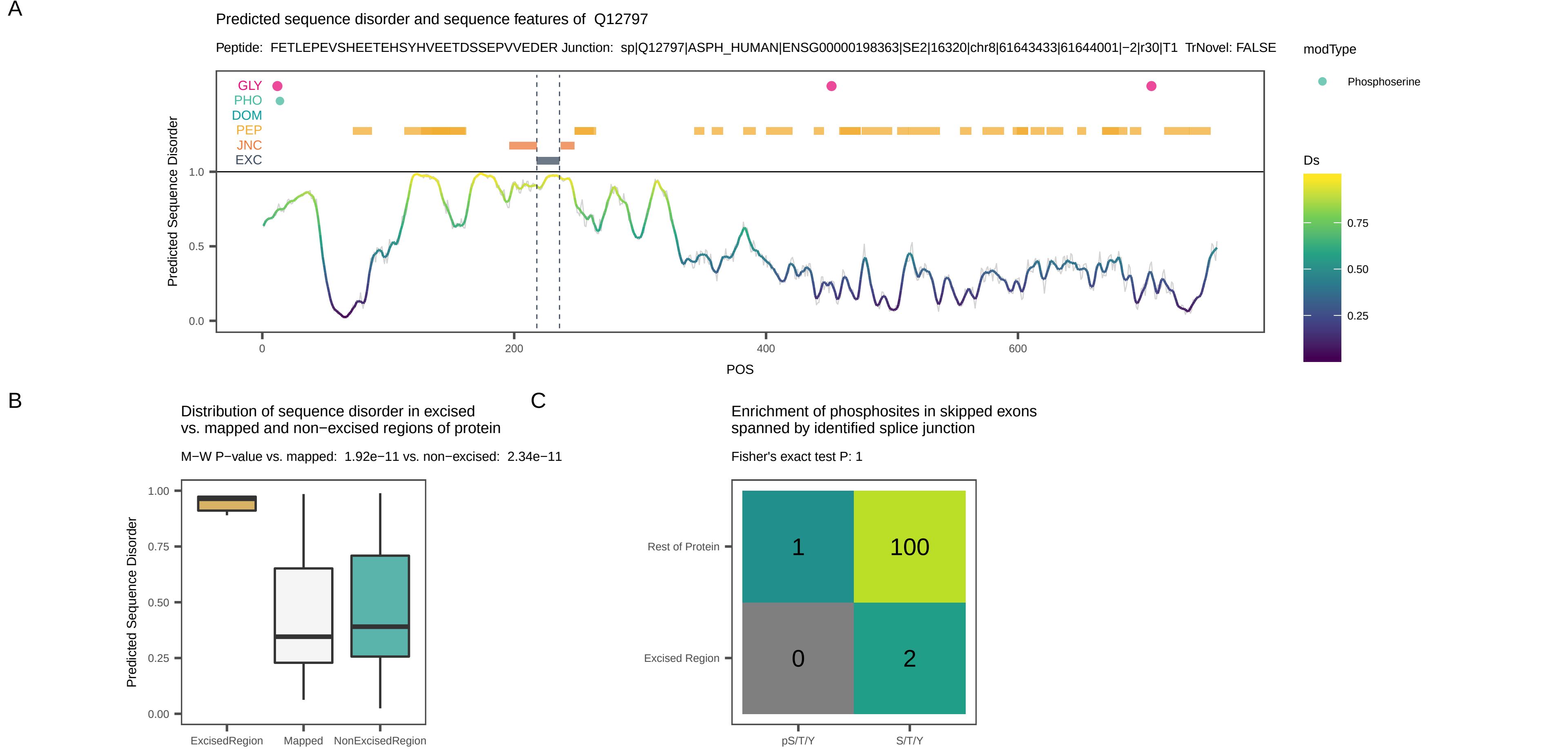
A
Predicted sequence disorder and sequence features of Q12797
Peptide: FETLEPEVSHEETEHSYHVEETDSSEPVVEDER Junction: sp|Q12797|ASPH_HUMAN|ENSG00000198363|SE2|16320|chr8|61643433|61644001|−2|r30|T1 TrNovel: FALSE
modType
Phosphoserine
GLY
PHO
DOM
PEP
JNC
EXC
Predicted Sequence Disorder
POS
Ds
B
Distribution of sequence disorder in excised vs. mapped and non−excised regions of protein
M−W P−value vs. mapped: 1.92e−11 vs. non−excised: 2.34e−11
Predicted Sequence Disorder
ExcisedRegion
Mapped
NonExcisedRegion
C
Enrichment of phosphosites in skipped exons spanned by identified splice junction
Fisher's exact test P: 1
Rest of Protein
Excised Region
pS/T/Y
S/T/Y
1
100
0
2
